# A simple and versatile method for frequent 24 h blood sample collection in healthy older adults

**DOI:** 10.1016/j.mex.2014.12.003

**Published:** 2014-12-26

**Authors:** A.A. Akintola, S.W. Jansen, R.B.P. Wilde, G. Hultzer, R. Rodenburg, D. van Heemst

**Affiliations:** a Department of Gerontology and Geriatrics, Leiden University Medical Centre, Leiden, The Netherlands; b Department of Surgery, Leiden University Medical Centre, Leiden, The Netherlands; c Intensive Care Department, Leiden University Medical Centre, Leiden, The Netherlands

**Keywords:** Aging, Repeated 24 h blood sampling, Sampling frequency, Hormones

## Abstract

Repeated 24 h blood sampling, which is required for time series analyses of metabolites and/or hormones that show strong fluctuations in blood concentration over time, has a higher failure rate in older adults. We tailored existing venipuncture protocols toward use for 24 h blood sampling (sampling frequency of 10 min) in older adults. The following modifications were made:

• Pre-sampling: evidence based risk assessment of older adults.
• During sampling:
  • Ultrasound-guided identification and characterisation of veins.
  • Use of 20-gauge arterial catheter with guide wire for venous access.
  • Measures to prevent and/or reduce unidirectional blood flow (fluid flow into but not out of the vein) included:
    • Use of hot water bottles to dilate veins.
    • Use of small gauge syringes, shortening of the extension line, and slowing of the blood withdrawal rate to reduce pressure on veins.
    • Stimulation of movement of the arm or retraction of the IV cannula to relieve mechanical flow obstruction.
• Post-sampling: prevention of bruising and prolonged bleeding.

## Graphical abstract

**Figure:**
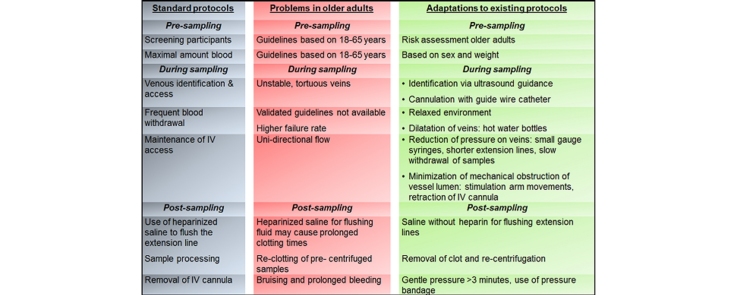

## Method details

Repeated 24 h blood sampling, a frequently used method in research, is required for time series analyses of metabolites and/or hormones that show strong fluctuations in blood concentration over time. Repeated 24 h blood sampling has a higher failure rate in older adults due to difficulty in establishing and maintaining venous access due to age- induced changes in the integrity of the skin, venous vasculature and valves. Therefore we customized pre-sampling, sampling and post-sampling procedures for continuous sampling in older adults. In our research center, the customized protocol was used for 24 h blood sampling with a sample frequency of 10 min in a group of 41 elderly mean (range) age of 66 (52–76) years. With the customized protocol, the mean (standard deviation) number of missing samples was 6.3 (7.0) out of 144 (4.4%). Thus, the customized protocol that is discussed in more detail below represents a useful and successful method for high frequency sampling of blood in healthy older adults.

## Pre-sampling

### Screening of participants

Maintenance of IV access and blood withdrawal proved to be most problematic in subjects older than 75 years of age, since venous capacitance and compliance reduces with age [1].

#### Standard protocols

According to the European guidelines [2], the age range for blood donation is 18–65 years. Above the age of 65 years, blood donation is allowed only at the discretion of the responsible physician [2]. This medical discretion can be applied on an individual basis or through a systematic approach based on an appropriate risk assessment.

#### Adaptations made

A systematic risk assessment (Table 1) by a suitably qualified individual (physician) was done before inclusion of older subjects (≥65 years) for continuous/repeated 24 h blood sampling.

##### Risks assessed a hard return

Medical status was assessed through medical history, medication use, physical examination and laboratory investigations. We assessed for:

- General state of health, presence of medical conditions and use of medications.
  - Acceptable systolic blood pressure was ≤180 mmHg and diastolic blood pressure ≤100 mmHg [2].
  - Acceptable pulse of 50–100 (regular) beats per minute [2].
  - Acceptable BMI of 18–30 kg/m^2^.
  - Absence of anemia.
- Assessment to exclude conditions that may interfere with maintenance of venous access such as:
  - Extensive scaring on one or both hands.
  - Previous mastectomy.
  - Previous fistula or vascular graft.
  - Severe arteriosclerosis.
  - Previous history of chemotherapy use.

### Determination of maximal amount of blood per sampling

There is presently no consensus as to the maximum amount of blood that can be withdrawn in older adults since blood donation guidelines are based on adults aged 18–65 years.

#### Standard protocols

Based on the European guideline [2] the maximum amount of blood that can be withdrawn is dependent on the weight and sex of the person, to a maximum of 500 ml over a 24 h period. No more than 15% of the estimated blood volume is to be collected as whole blood, because of the risk of adverse reactions [2].

#### Adaptations made

We calculated maximum blood volume that can be withdrawn based on the weight, height, age and gender using a validated formula developed by the International Council of Standardisation in Haematology (ICHS) [3].

## During sampling

### Venous cannulation: identification

In older adults, skin loses tone and elasticity and becomes more fragile and prone to bruising. Upon finding a suitable blood withdrawal site, loss of subcutaneous tissue in older adults result in their veins being less stable, less visible, prone to receding and rolling under the skin thus reducing available IV access sites.

#### Standard protocols

Standard venepuncture and phlebotomy guidelines involve visual identification of forearm veins (median cubittal and median veins) followed by venepuncture [4], [5].

#### Adaptations made

Ultrasonography (US) was used for peripheral vein cannulation in subjects with difficult venous access to identify the peripheral vessels and guide the cannulation of the peripheral vein [6], [7]. US guidance was used for:

- Easier localisation of the vessel and its relation to surrounding anatomical structures.
- Determination of vascular quality and tortuosity of veins.
- Presence and location of intravascular valves.

### Venous cannulation: access

In older adults more time is needed to find the most appropriate access site, since veins are more difficult to find, more tortuous and veins have a tendency to collapse more due to degeneration of the vascular wall [8].

#### Standard protocols

Inspection of the antecubital fossa in preparation for cannulation, skin preparation with 2% chlorhexidine and insertion of appropriate cannula. Pressure is applied in cases of failed cannulation.

#### Adaptations made

In addition to the standard protocol, the following adaptations were implemented to improve success in older adults:

i. US-guided identification of the cephalic vein, basilica vein and median cubital vein.
ii. Cannulation with a 20-gauge guide wire catheter, 8 cm in length, inserted with Seldinger technique (Arrow International, Reading, PA, USA).
  - ! The choice to use arterial catheters is supported by literature reporting that the use of standard-length (3–5 cm) catheters positioned in the deep brachial or basilica vein is frequently complicated by their dislodgment or dislocation [7]. Furthermore, the use of a longer IV-catheter provides freer arm movement and will increase comfort in individual subjects. Moreover, the amount of catheter failure and dislocation compared to standard-length IV catheters in the deep brachial and basilica vein is lower [6], [7]. Keyes et al. [6] observed that the failure rate of peripherally inserted catheters was 8% within the first hour after venous cannulation. In a recent study, Elia et al. reported percentages of catheter failure of 45% vs. 14%, [RR 3.2 (95% CI 1.4–7.3)], and dislocation of 42.5% vs. 2.3% [RR 18.7 (95% CI 2.0–134.2)] when comparing standard-length to long IV catheters, inserted in the deep brachial and basilica vein [7].
  - ! Nicking the skin with a scalpel or the use of a dilatator is not necessary.
  - Venepuncture and catheter introduction is an aseptic procedure necessitating the use of sterile gloves. Finger guidance of the shaft of the exposed needle is not required.
iii. In subjects with prominent veins, the basilica vein was the preferred access route because the basilica vein has a lager diameter compared with the cephalic vein, is easier to access and more suited for frequent blood sampling [9]. We cannulated with Arrow Catheterization Set (Product No. SAC 00820) without complications.

### Frequent blood withdrawal

#### Standard protocols

No published validated protocol was found for older adults. A previous study recommended addition of heparin 100 IU/mL continuous saline infusion to prevent the IV system from clots and to reduce the number of catheter-related phlebitis/occlusions [10].

#### Adaptations made

**Fig. 1:**
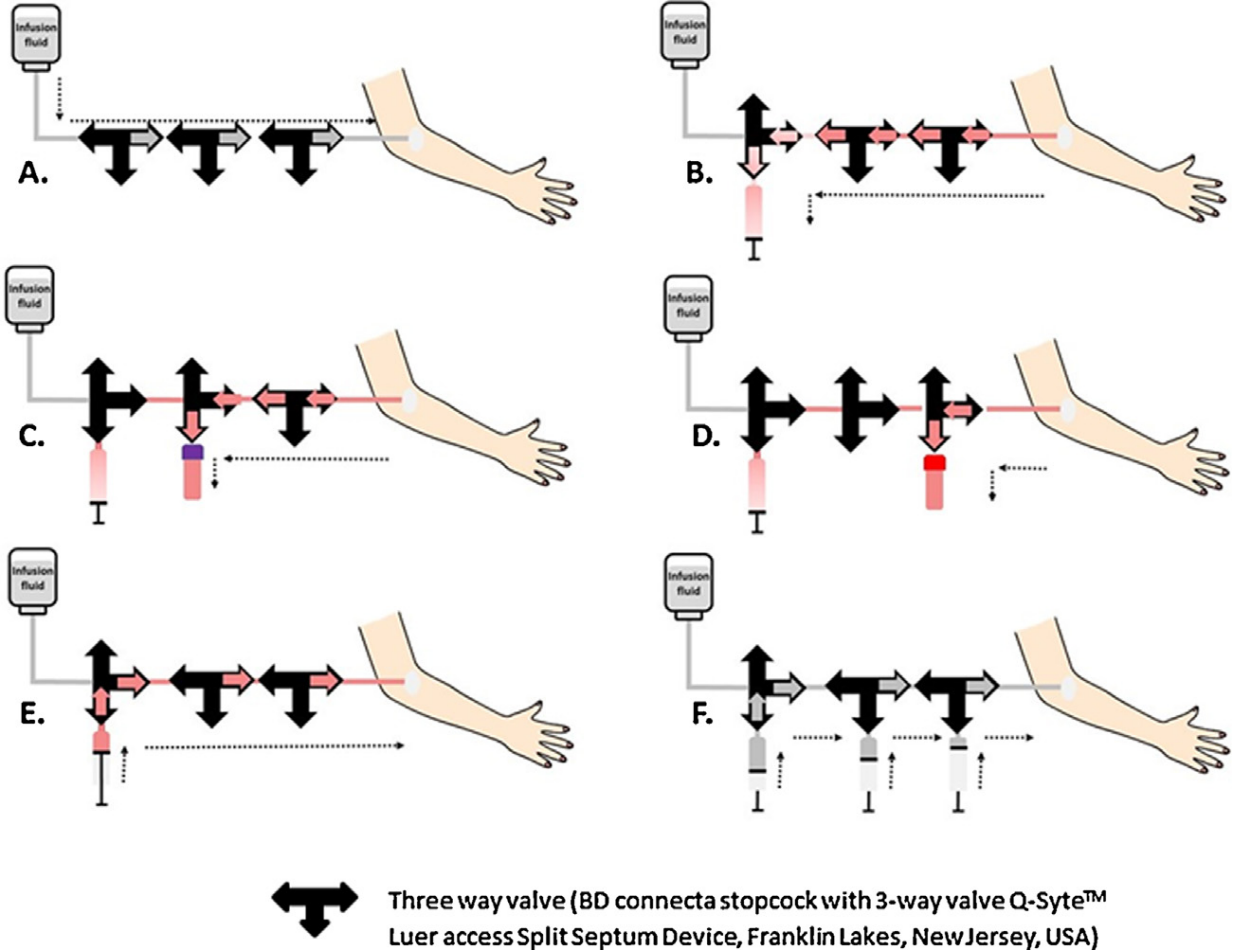
Schematic overview of a closed method for frequent 24 h blood sample collection. (A) Continuous infusion. (B) Turn the left three way valve 90°, withdraw 5 ml of saline/heparin mixed with blood. (C) Turn the middle three way valve 90°, withdraw EDTA sample and place it directly on ice. (D) Turn the right three way valve 90°, withdraw serum sample and let it clot for at least 30 min. (E) Turn the right and middle three way valve 90° and empty the syringe filled with the saline/heparin mixed with blood. (F) Flush with saline and turn the left three way valve 90° and continue with infusion (return to position (A)).

For detailed hormonal and metabolic profiling of older adults aged 55–78 years, we aimed at total blood withdrawal of 432 ml over a 24 h period. Serum (2 ml) and plasma (1.2 ml) samples were withdrawn every 10 min, with replacement by 480 ml of heparinized saline, using the following protocol (Fig. 1):

i Continuous infusion of heparinized saline (0.9% NaCl).
ii For the preparation of the heparinized saline, 500 IU of heparin was added to 500 ml of saline. This was infused over 24 h via an infusion pump at a rate of 20 ml h^−1^.
iii Withdrawal of 5 ml of saline/heparin mixed with blood, without disconnecting the syringe from the blood withdrawal system.
iv Placement of the 1.2 ml ethylenediaminetetraacetic acid (EDTA) Sarstedt S-monovette^®^ (Nümbrecht, Germany) on the multiadaptor for S-monovette^®^ (Nümbrecht, Germany) for blood sample withdrawal after which the blood was mixed gently with the EDTA and placed immediately on ice.
v Placement of the 2 ml blood collection clotting tube BD (Franklin Lakes, USA) on the BD vacutainer^®^, (Franklin Lakes, USA). The sample was withdrawn and mixed with the clotting activator by gently turning the tube five times, after which it was allowed to clot for at least 30 min at room temperature.
vi Flushing of the blood from the 5 ml syringe back into the subject, to reduce the total amount of blood that will be withdrawn.
vii Flushing of the blood withdrawal system (including the extension line) to remove diluted blood, using 5 ml saline (0.9% NaCl).

### Maintenance of IV access

In older adults, blood sampling was sometimes jeopardised by unidirectional blood flow (free flow of fluid into the vein but not out of the vein), with resultant impedance of sustained blood withdrawal. This is possibly due to reduced tone of the vessel wall, age- induced fibrosis of the wall of the veins and valve leaflets.

### Standard protocols

No validated guidelines were available for older subjects.

### Adaptations made

- Creation of a relaxing enviroment to reduce stress for the participant.
- The participants were allowed to move their arm freely.
- Application of hot water bottles to increase the diameter of the vessel.
- Use of small gauge syringes (e.g., 2 ml syringes instead of 5 ml syringes) to reduce pressure on the vessel wall.
- Obtaining blood samples very slowly e.g., using 1 ml/2ml syringes.
- Retraction of the IV cathether a few milimeters to change its position.

## Post sampling

### Processing of samples

Variable, sometimes prolonged clotting times in older adults.

#### Standard protocols

There are different protocols depending on the hormone to be measured.

#### Adaptations made

For serum samples, blood was allowed to clot. Clotting time was very variable for older adults, ranging from approximately 15–70 min. Preferably within 60 min of sampling, tubes were centrifuged at 4000 rpm at 4 °C for 10 min. Because of the clotting problems, re-clotting sometimes occurred in the serum samples after centrifuging. This was managed by manual removal of the clot from the sample tube followed by re-centrifuging.

After centrifuging, serum and plasma were pipetted into 500 μl Microvettes^®^ Sarstedt (Nümbrecht, Germany), which were then stored first at −20 °C and transferred to −80 °C within 24 h of blood withdrawal. Once frozen, samples were not allowed to thaw until laboratory analysis.

### Removal of IV cannula

Prolonged bleeding, bruising.

#### Standard protocols

The WHO guidelines on drawing blood recommend inspecting the puncture site and if bleeding occurs then applying gentle pressure on the puncture site until bleeding has stopped. If no bleeding occurred it is recommended to apply a bandage [4].

#### Adaptations made

For the prevention of bruising we applied gentle pressure to the puncture site for at least one minute. Thereafter participants were asked to apply gentle pressure for at least 2 min. A second inspection was made to check for bleeding; if bleeding occurred, gentle pressure was continued; if not then a bandage was placed. Extra attention was paid to older adults using anti-thrombotic medications.

## Figures and Tables

**Table 1:** Schematic overview of systematic assessment of older adults, to determine eligibility for frequent blood sampling.

Standard screening	Pay attention to	Reason for attention
Medical history	Previous contra-indications to blood donation	Contra-indications of placement of IV cannula
Previous difficulty with venipuncture	Frail veins, stiffened valves
Previous mastectomy/relevant surgery	
Previous fistula or vascular graft	
Severe arteriosclerosis	
History of chemotherapy	

Medication use	Anti-coagulants	Increased bleeding risk causing bruises
Medications relevant to hormone(s) of interest	

Review of systems	Palpitations	These symptoms are indicators of underlying cardiac and brain hypo-perfusion, withdrawal of high amounts of blood may lead to damage to those tissue due to decreased oxygenation
Chest pain
Signs of TIA (neurological paralysis)

Medical examination	Appearance of blood vessels	Problems with insertion of IV cannula
Extensive scaring on one or both hands	
Pulse	To detect unknown cardiac problems
Blood pressure	
Cardiac sounds	
